# A significant proportion of venous thromboembolism events in general surgical patients occurs after discharge: analysis of the ACS-NSQIP Essentials database

**DOI:** 10.1186/s13741-019-0131-1

**Published:** 2019-12-13

**Authors:** Abdelghaffar K. Salous, Ashraf Reyad, Karen Sweeney, Arun Mavanur

**Affiliations:** 1 Department of General Surgery, Sinai Hospital of Baltimore, Baltimore, MD USA; 2 Department of Plastic and Reconstructive Surgery, Johns Hopkins School of Medicine, 720 Rutland Avenue, Rm 749A, Baltimore, MD 21205 USA

**Keywords:** Venous thromboembolism, Pulmonary embolism, Chemoprophylaxis, NSQIP

## Abstract

**Background:**

Venous thromboembolism (VTE) is a major cause of morbidity and mortality in general surgical patients.

**Methods:**

The ACS-NSQIP database was queried and VTE data were collected and analyzed to assess the incidence of VTE at a 500-bed, non-profit, teaching, inner city, community hospital and similar peer institutions from January 1, 2006 to December 31, 2011.

**Results:**

Post-discharge VTE events accounted for 40% of all VTE events within 30 days of discharge. Data show a significant proportion of post-discharge VTE events that may be preventable with extending VTE prophylaxis in the post-discharge period.

**Conclusion:**

This is the first paper to report on this high post-discharge incidence of VTE in general surgical patients and to recommend continuation of VTE prophylaxis in the post-discharge period.

## Background

Venous thromboembolism (VTE) is a complex multifactorial disease entity associated with significant morbidity and mortality, with two main manifestations: deep venous thrombosis (DVT) and/or concomitant pulmonary embolism (PE). The major risk factors (Heit, 2015), the preventive measures, and therapeutic modalities (Thaler et al., 2015) for VTE have been extensively reviewed. The economic burden associated with VTE is significant, 1.5-fold higher for the surgical patient. The greatest difference in the cost of VTE management is within the first 3 months after incidence (Cohoon et al., 2015).

The best available data regarding the chronological incidence of VTE were described in the Worcester retrospective population study in Olmsted, MN, which examined the incidence of VTE from 1981 to 2000. In that study, the annual incidence of venous thromboembolism increased with age and was 117 per 100,000 patient-years (Silverstein et al., 1998). A follow-up of the Worcester study (2001–2009) confirmed this observation (Huang et al., 2014). A number of interesting trends were confirmed by these studies. First, the risk of developing VTE was 100-fold higher in hospitalized patients. Second, VTE events with hospitalization for surgery accounted for 24% of these cases (Heit et al., 2002). Finally, although the cumulative incidence of VTE increased steadily over time from 1.6% at 7 days to 30.4% at 10 years in these patients, the risk of the first recurrence was highest in the first 6-12 months (Heit et al., 2000).

The impact of VTE on morbidity, mortality and economic cost understandably led to its adoption as an American College of Surgeons National Surgical Quality Improvement Program (ACS-NSQIP) quality measure, stimulated the development of VTE risk stratification tools such as the Caprini risk assessment and accelerated implementation of such tools by several institutions for inpatient VTE chemoprophylaxis. Yet while reduction of inpatient VTE events is tangible in healthcare institutions adopting such measures, this finding is probably shortsighted. In other words, the perceived benefits of decreased pre-discharge VTE events may be offset or even be eclipsed by the rise in post-discharge VTE events. Data about the incidence of VTE in the immediate and delayed postoperative period, although tracked by ACS-NSQIP, are presently under-analyzed. In this light, this paper is the first to explore this question, aided by the ACS-NSQIP database and closer examination of our patient population.

## Methods

The ACS-NSQIP database was queried for pre-discharge and post-discharge VTE events for surgical patients at a community-based medium-size hospital and peer institutions nationwide. Data included all surgical patients from January 1, 2006 to December 31, 2011 (Table 1), who were hospitalized for surgery and followed up within 30 days after discharge. Patients who had clinical VTE events were reported. The proportion of VTE events, breakdown into PE and DVT events, was calculated and reported using Microsoft Excel.

**Table 1:** Total number of patients included in the analysis. Data were downloaded from the ACS-NSQIP database from January 1, 2006 to December 31, 2011 for all patients who were hospitalized for surgery and followed up within 30 days after discharge

Group	Pre-discharge	Post-discharge
NSQIP VTE	10969	7251
Sinai VTE	43	32
NSQIP DVT	7733	4645
Sinai DVT	29	18
NSQIP PE	3236	2606
Sinai PE	14	14

Since the ACS-NSQIP Essentials database does not collect specific procedural patient details such as Caprini score, we utilized our electronic health record to review the characteristics of the cohort of these patients, who were concurrently reported in the ACS-NSQIP database in the same time period. We were specifically interested in identifying a cutoff Caprini value to use as a guideline to recommend extending VTE prophylaxis following discharge.

## Results

**Fig. 1:**
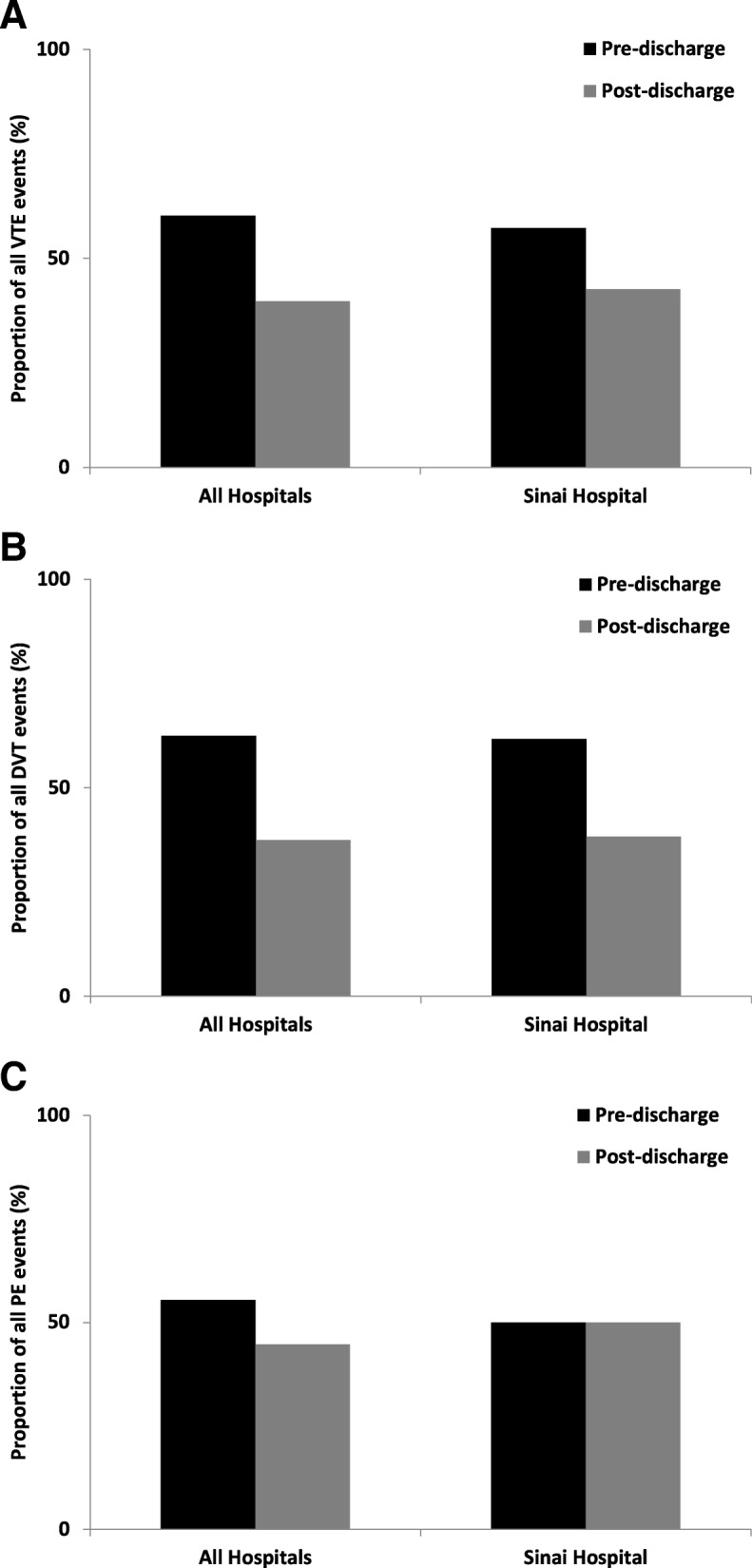
**a** The percentage of pre-discharge and post-discharge VTE events during hospitalization and within 30 days of postoperative follow-up for Sinai hospital and equivalent peer institutions. **b**, **c** Breakdown of VTE events into DVT and PE subcategories. Data derived from NSQIP database for medium-size community hospitals from January 1, 2006 to December 31, 2011

Post-discharge VTE events approximately accounted for 40% all VTE events within 30 days of discharge (Fig. 1a). VTE events were subclassified into DVT and PE events. A slightly higher fraction of PE events (45%) was detected upon post-discharge follow-up than DVT events (38%) (Fig. 1b, c).

**Fig. 2:**
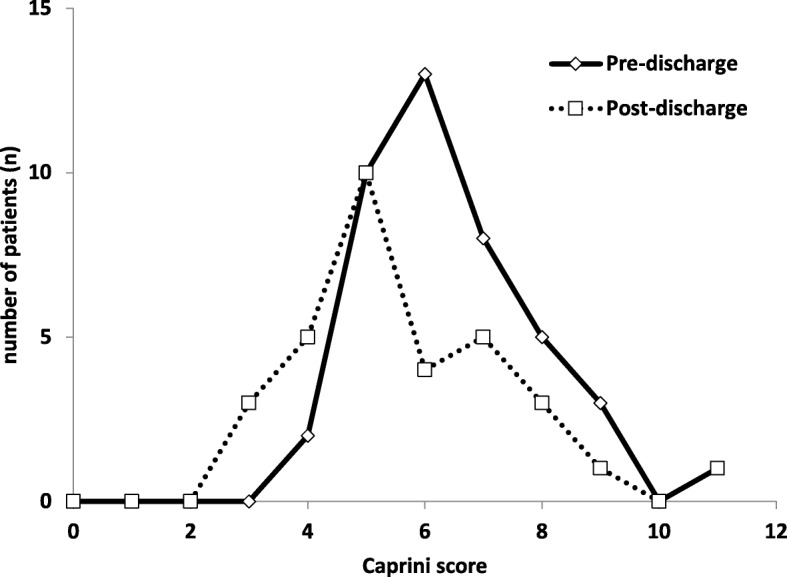
Distribution of VTE events in surgical pre-discharges (diamonds) and post-discharges (squares). The mean Caprini score was 6.4 for pre-discharges (43 total) and 5.7 for post-discharges (32 total). The distributions appeared somewhat normal

Since the incidence of VTE, DVT, and PE was similar in our hospital and the aggregate data for other peer institutions participating in the ACS-NSQIP database, we examined VTE incidents in relation to the Caprini score in our hospital population, as discussed in the methods section (Fig. 2). While both patient populations had a fairly similar distribution, the mean Caprini score was 5.7 for patients following discharge, compared to 6.4 for the pre-discharge inpatient population. The data distribution profiles appeared somewhat similar, suggesting the measures of central tendency may be shifted even more with extended prophylaxis.

## Discussion

Several layers of complexity present a challenge to addressing the prophylaxis of VTE in surgical patients. First, the incidence of VTE is increased with comorbidities and a significant economic burden. Second, this is further compounded by the lack of clear data about the incidence and chronologic variation of VTE. In turn, this casts doubt on the adequacy of treatment in terms of duration. Querying the ACS-NSQIP database about the incidence of VTE in surgical patients surprisingly showed 40% of VTE events occurred within 30 days post-discharge. While it is reasonable to presume that the vast majority of patients received VTE chemoprophylaxis in the pre-discharge setting, the true incidence of post-discharge VTE events remains unknown and at best, significantly understated. This assertion resonates with the trends in the Worcester study and the limited post-discharge surveillance to 30 days.

This finding strongly suggests that extending VTE prophylaxis after discharge would significantly decrease the proportion of post-discharge VTE events with subsequent associated decreases in morbidity and mortality. The orthopedic surgery and surgical oncology literature lend strong support to this proposition. The prevalence of VTE events across surgical specialties was extensively reviewed and brought to light the high VTE rate in orthopedic surgery patients, ~40–60%, compared to general surgery patients, ~15-40% (Geerts et al., 2004). Additionally, randomized controlled clinical trials showed that the rate of VTE events significantly decreased in orthopedic surgery patients who remained on extended VTE prophylaxis for 30 days after their surgery (Falck-Ytter et al., 2012). Current guidelines recommend using one of several agents for VTE prophylaxis for patients undergoing major orthopedic surgery (grade 1B). The incidence of VTE events in orthopedic surgery patients who continued extended VTE chemoprophylaxis for 30 days was 1.5% compared to 4.5% and appeared to reach a plateau level after that time point (Falck-Ytter et al., 2012). Two strong studies in surgical oncology echo these findings. A landmark study by Bergqvist et al. in the New England Journal of Medicine also examined the use of subcutaneous enoxaparin for 4 weeks in cancer patients who underwent abdominal surgery for cancer and showed a significant decrease in VTE events (Bergqvist et al., 2002). Another randomized well-controlled multi-center clinical trial corroborated this conclusion (Rasmussen et al., 2006). Taking all these results in consideration, we advocate extending VTE prophylaxis in general surgical patients at high risk for VTE events.

To aid in patient selection, we proceeded to analyzing our patient population at Sinai hospital with respect to the Caprini score for several reasons. First, the proportion of VTE events and their breakdown categories were strikingly similar to the larger aggregate sample from the ACS-NSQIP database. Second, the ACS-NSQIP Essentials database does not report risk factors or measures such as Caprini score, which were readily available for our patients. Third, Caprini score served as a uniform risk measure. Taken together, our findings should be cautiously extrapolated to the larger population of surgical patients. Building on the results discussed here, we propose to use a Caprini score of 4 as a cutoff value to extend VTE prophylaxis for 4 weeks in general surgical patients to balance the risks of VTE events and bleeding. We believe our results and proposition pave the way for studying VTE incidence in general surgical patients in randomized controlled prospective clinical trials. Subsequently, this would help revise guidelines for secondary appropriate prophylaxis for high-risk patients and drive reduction in morbidity and mortality.

## Conclusion

Analysis of data reported in ACS-NSQIP reveals 40% of VTE events in general surgical patients occur after discharge. Closer examination of a representative sample of such patients post-discharge at our hospital shows a relatively high Caprini score. This paper is the first to report the high incidence of VTE events in general surgical patient post-discharge and the first to recommend extending VTE prophylaxis for 30 days after discharge for the high-risk surgical patient, defined as one with a Caprini score ≥4. The application of this guideline showed that a significant impact on other patients is likewise projected to reduce the mortality, morbidity, and economic burden of VTE in surgical patients.

## Data Availability

Data were downloaded from ACS-NSQIP database; please refer to the methods section for details.

## Abbreviations

VTE: Venous thromboembolism
PE: Pulmonary embolism
DVT: Deep venous thrombosis
